# Comparative Transcriptome Analysis Identifies CCDC80 as a Novel Gene Associated with Pulmonary Arterial Hypertension

**DOI:** 10.3389/fphar.2016.00142

**Published:** 2016-06-07

**Authors:** Shota Sasagawa, Yuhei Nishimura, Hirofumi Sawada, Erquan Zhang, Shiko Okabe, Soichiro Murakami, Yoshifumi Ashikawa, Mizuki Yuge, Koki Kawaguchi, Reiko Kawase, Yoshihide Mitani, Kazuo Maruyama, Toshio Tanaka

**Affiliations:** ^1^Department of Molecular and Cellular Pharmacology, Pharmacogenomics and Pharmacoinformatics, Mie University Graduate School of Medicine, TsuJapan; ^2^Mie University Medical Zebrafish Research Center, TsuJapan; ^3^Department of Systems Pharmacology, Mie University Graduate School of Medicine, TsuJapan; ^4^Department of Omics Medicine, Mie University Industrial Technology Innovation Institute, TsuJapan; ^5^Department of Bioinformatics, Mie University Life Science Research Center, TsuJapan; ^6^Department of Anesthesiology and Critical Care Medicine, Mie University Graduate School of Medicine, TsuJapan; ^7^Department of Pediatrics, Mie University Graduate School of Medicine, TsuJapan

**Keywords:** pulmonary arterial hypertension, comparative transcriptome analysis, weighted gene co-expression network analysis, CCDC80, EDN1, COL1A1, systems pharmacology

## Abstract

Pulmonary arterial hypertension (PAH) is a heterogeneous disorder associated with a progressive increase in pulmonary artery resistance and pressure. Although various therapies have been developed, the 5-year survival rate of PAH patients remains low. There is thus an important need to identify novel genes that are commonly dysregulated in PAH of various etiologies and could be used as biomarkers and/or therapeutic targets. In this study, we performed comparative transcriptome analysis of five mammalian PAH datasets downloaded from a public database. We identified 228 differentially expressed genes (DEGs) from a rat PAH model caused by inhibition of vascular endothelial growth factor receptor under hypoxic conditions, 379 DEGs from a mouse PAH model associated with systemic sclerosis, 850 DEGs from a mouse PAH model associated with schistosomiasis, 1598 DEGs from one cohort of human PAH patients, and 4260 DEGs from a second cohort of human PAH patients. Gene-by-gene comparison identified four genes that were differentially upregulated or downregulated in parallel in all five sets of DEGs. Expression of coiled-coil domain containing 80 (*CCDC80*) and anterior gradient two genes was significantly increased in the five datasets, whereas expression of SMAD family member six and granzyme A was significantly decreased. Weighted gene co-expression network analysis revealed a connection between *CCDC80* and collagen type I alpha 1 (*COL1A1*) expression. To validate the function of CCDC80 *in vivo*, we knocked out *ccdc80* in zebrafish using the clustered regularly interspaced short palindromic repeats (CRISPR)/Cas9 system. *In vivo* imaging of zebrafish expressing a fluorescent protein in endothelial cells showed that *ccdc80* deletion significantly increased the diameter of the ventral artery, a vessel supplying blood to the gills. We also demonstrated that expression of *col1a1* and endothelin-1 mRNA was significantly decreased in the *ccdc80*-knockout zebrafish. Finally, we examined Ccdc80 immunoreactivity in a rat PAHmodel and found increased expression in the hypertrophied media and adventitia of the pre-acinar pulmonary arteries (PAs) and in the thickened intima, media, and adventitia of the obstructed intra-acinar PAs. These results suggest that increased expression of CCDC80 may be involved in the pathogenesis of PAH, potentially by modulating the expression of endothelin-1 and *COL1A1*.

## Introduction

Pulmonary arterial hypertension is a progressive disease characterized by increased pulmonary vascular resistance due to vasoconstriction and remodeling (reviewed in Pullamsetti et al., 2014; McLaughlin et al., 2015). The pathologic abnormalities in vascular lesions include intimal hyperplasia, medial thickness, plexiform lesions, and thrombosis *in situ* (Iwashita et al., 2014; Otsuki et al., 2015; Shinohara et al., 2015), which are caused by increased migration and proliferation of smooth muscle cells and adventitial fibroblasts, abnormal endothelial cell proliferation, and impaired apoptosis. Although several treatment options have become available and have significantly improved morbidity and mortality, the 5-year survival rate for PAH patients remains at ~60% (Korsholm et al., 2015). Early diagnosis and accurate prognostic stratification of patients at baseline and during follow-up are important to ensure optimal therapeutic strategies (Pezzuto et al., 2015). Thus, finding novel genes involved in the pathogenesis of PAH could provide a better understanding of the pathophysiological mechanisms and suggest novel therapeutic approaches for the disease (Guignabert et al., 2015; Machado et al., 2015).

Transcriptome analysis could represent a new frontier in the search for novel biomarkers and/or therapeutic targets in various diseases, because it facilitates the identification of panels of genes specifically dysregulated in affected tissues (Nishimura et al., 2007, 2015b; Oldham et al., 2008; Oka et al., 2010; Sasagawa et al., 2016). A number of transcriptome analyses of PAH patients and PAH animal models have been performed and the data have been deposited in a public database (Barrett et al., 2009). These include data derived from two cohorts of human patients (Mura et al., 2012; Zhao Y. et al., 2014; Zhao Y.D. et al., 2014); a rat PAH model caused by treatment with the vascular endothelial growth factor receptor inhibitor SU5416 under conditions of hypoxia (Moreno-Vinasco et al., 2008); a mouse PAH model caused by overexpression of Fra-2 (Biasin et al., 2014), a causative gene for systemic sclerosis; a mouse PAH model caused by schistosomiasis (Graham et al., 2013); a rat model caused by left heart disease (Hoffmann et al., 2011); a rat model caused by *Pneumocystis* infection (Swain et al., 2014); and a mouse PAH model caused by deletion of cavin-1 (Swärd et al., 2013). In this study, we sought to identify genes commonly dysregulated in PAH in both human and rodent models. Therefore, we selected for analysis both cohorts of human PAH patients (Mura et al., 2012; Zhao Y. et al., 2014; Zhao Y.D. et al., 2014); two mouse models caused by schistosomiasis (Graham et al., 2013) and Fra-2 overexpression (Biasin et al., 2014), which were selected because schistosomiasis and connective tissue diseases such as systemic sclerosis are major causes of PAH (Simonneau et al., 2013); and a rat PAH model caused by SU5416 and hypoxia (Moreno-Vinasco et al., 2008), which we included in this study because we have successfully employed this PAH model (Otsuki et al., 2015; Shinohara et al., 2015). We acknowledg that our transcriptome analysis of these datasets may not detect genes involved in other common causes of PAH, such as left heart and/or lung diseases.

We performed a comparative transcriptome analysis of the two human and three rodent PAH datasets and found that coiled-coil domain containing 80 (CCDC80) may be a novel biomarker and therapeutic target in PAH. We validated the function of CCDC80 as it relates to PAH using zebrafish. Many transgenic zebrafish lines expressing fluorescent proteins in various cells have been developed and are available through public resources, enabling multiple approaches to *in vivo* imaging of the relevant target cells in zebrafish larvae (Nishimura et al., 2015a). Specific genes can also be knocked out using clustered regularly interspaced short palindromic repeats (CRISPR)-Cas9 systems (Auer and Del Bene, 2014). Thus, when used in combination with advanced technologies for genetic manipulation, *in vivo* imaging of the zebrafish ventral artery, which supplies blood to the gill arches where gas exchange occurs by diffusion (Jonz and Nurse, 2008), may be a useful tool to characterize the function of genes related to PAH. Our *in vivo* validation using the zebrafish model revealed that *ccdc80* knockout increased the diameter of the ventral artery. Finally, we examined Ccdc80 expression in a rat PAH model and found increased staining in the hypertrophied media and adventitia of the pre-acinar pulmonary arteries (PAs) and in the thickened intima, media, and adventitia of the obstructed intra-acinar PAs.

## Materials and Methods

### Ethics Statement

This study was carried out in strict accordance with Japanese law [The Humane Treatment and Management of Animals (2014), Standards Relating to the Care and Management of Laboratory Animals and Relief of Pain (2013), and the Guidelines for Proper Conduct of Animal Experiments (2006) (Science Council of Japan, 2006; Ministry of the Environment Japan, 2013, 2014)]. All efforts were made to minimize animal suffering. Mie University Institutional Animal Care and Use Committee guidelines state that no approval is required for experiments using zebrafish.

### Compounds

Sodium nitroprusside (SNP) was obtained from Millipore (Billerica, MA, USA) and dissolved in 0.3x Danieau’s solution (19.3 mM NaCl, 0.23 mM KCl, 0.13 mM MgSO_4_, 0.2 mM Ca(NO_3_)_2_, 1.7 mM HEPES, pH 7.2) immediately before use in experiments. KT5823 was obtained from Tocris (Bristol, UK). Stock solutions of KT5823 were prepared by dissolving in dimethyl sulfoxide (Nacalai Tesque, Kyoto, Japan). L-NAME was obtained from Dojin Chemicals (Kumamoto, Japan). 2-Phenoxyethanol was obtained from Wako Chemicals (Osaka, Japan).

### Comparative Transcriptome Analysis

To compare DEGs in human PAH and rodent models of PAH of different etiologies, we analyzed five lung transcriptome datasets. GSE8078 was derived from a rat PAH model caused by treatment with the VEGF receptor inhibitor SU5416 under conditions of hypoxia (Moreno-Vinasco et al., 2008). Adult male rats received a single subcutaneous injection of SU-5416 and were housed under hypoxic condition for 3.5 weeks. mRNA was isolated from whole lung tissue of the PAH rats (*n* = 3) and the control rats (*n* = 4). GSE51222 was derived from a mouse PAH model caused by overexpression of Fra-2 (Biasin et al., 2014), and mRNA was isolated from the lung homogenates of 8-week-old transgenic mice (*n* = 3) and wild-type littermate controls (*n* = 3). GSE48936 was derived from a mouse PAH model caused by schistosomiasis (Graham et al., 2013). Female mice received an intraperitoneal injection of *Schistosoma mansoni* eggs followed 2 weeks later by challenge with *S. mansoni* eggs injected via the tail vein. One week later, lung mRNA was isolated from injected (*n* = 3) and uninjected (*n* = 3) mice. GSE24988 and GSE53408 were derived from human PAH patients (Mura et al., 2012; Zhao Y. et al., 2014; Zhao Y.D. et al., 2014). For our study, we compared the transcriptomes of patients with pulmonary fibrosis without PAH (*n* = 22) and with intermittent PAH (*n* = 45) in GSE24988 and controls (*n* = 11) and patients with PAH (*n* = 12) in GSE53408.

The raw data were normalized using the packages “affy” (Gautier et al., 2004) for GSE8078, “limma” (Ritchie et al., 2015) for GSE51222, and “oligo” (Carvalho and Irizarry, 2010) for GSE48936, GSE24988, and GSE53408 in Bioconductor (Gentleman et al., 2004). Probes with reliable signals were selected and subjected to RankProd (Breitling et al., 2004) analysis to identify DEGs in the PAH groups compared with control groups in each dataset. False discovery rates were set at 20% for the rodent PAH models, 10% for one human cohort (GSE24988), and 5% for the second human cohort (GSE53408). The gene symbols of the DEGs were converted to the human orthologs using Life Science Knowledge Bank (World Fusion, Tokyo, Japan), and UniProt IDs were added using the ID mapping tool (UniProt Consortium, 2015). The lists of DEGs are shown in Supplementary Tables S1-1–S1-5. The DEGs common to all five PAH transcriptome datasets are shown in **Table 1**.

**Table 1:** Differentially expressed genes common to the five PAH transcriptome datasets.

		SU5416/hypoxia GSE8078		Fra-2 TG GSE51222		Schistosomiasis GSE48936		Human PAH GSE24988		Human PAH GSE53408	
Symbol	UniProt ID	log (PAH/C)	FDR	log (PAH/C)	FDR	log (PAH/C)	FDR	log (PAH/C)	FDR	log (PAH/C)	FDR
*AGR2*	095994	1.17	0.01	0.87	0.01	1.26	0.03	0.35	0.00	1.19	0.00
*CCDC80*	Q76M96	0.71	0.14	0.61	0.06	1.02	0.08	0.17	0.00	1.15	0.00
*GZMA*	P12544	–0.61	0.06	–4.18	0.00	–0.77	0.17	–0.16	0.04	–0.71	0.00
*SMAD6*	043541	–1.12	0.00	–0.51	0.16	–1.18	0.03	–0.03	0.10	–0.96	0.00

### Weighted Gene Co-Expression Network Analysis

To identify molecular interactions between genes potentially related to PAH, we performed weighted gene co-expression network analysis (WGCNA). WGCNA can organize transcriptomic data into networks based on underlying expression relationships, such as correlations, to elucidate novel connections between genes (Oldham et al., 2008). Expression data for 40 genes commonly dysregulated in the five PAH transcriptome datasets (Supplementary Table S2) were subjected to WGCNA (Langfelder and Horvath, 2008) in Bioconductor (Gentleman et al., 2004). The 40 genes were clustered into three modules of correlated genes: the first contained *CCDC80*, the second contained both *SMAD6* and *GZMA*, and the third contained *AGR2*. The gene networks in modules 1, 2, and 3 were identified using thresholds of 0.05, 0.25, and 0.02, respectively.

### Zebrafish Strains

We used two lines of zebrafish: a Tg (fli1:EGFP) line (Lawson and Weinstein, 2002) obtained from Zebrafish International Resource Center (Eugene, OR, USA) (Varga, 2011) and an albino line (Kelsh et al., 1996) obtained from the Max Planck Institute for Developmental Biology (Tübingen, Germany). Zebrafish were bred and maintained according to previously described methods (Westerfield, 2007; Nishimura et al., 2016). Briefly, zebrafish were raised at 28.5°C ± 0.5°C with a 14 h/10 h light/dark cycle. Embryos were obtained by natural mating and cultured in 0.3x Danieau’s solution until 6 dpf, at which time they were used for the *in vivo* imaging analyses or were processed for quantitative polymerase chain reaction (qPCR).

### Knockout of ccdc80 in Zebrafish

Knockout of ccdc80 in zebrafish was performed by the ready-to-use CRISPR/Cas9 method (Kotani et al., 2015). CRISPR RNA (crRNA) targeting a 5′-AAGCAGCCCGACCGATAAAC-3′ sequence in the ccdc80 genome and trans-activating crRNA (tracrRNA; Kotani et al., 2015) were obtained from FASMAC (Kanagawa, Japan). Recombinant Cas9 protein was obtained from Toolgen (Seoul, South Korea). In brief, crRNA, tracrRNA, and Cas9 protein were dissolved in sterilized water at concentrations of 250, 1000, and 1000 ng/μl, respectively, and stored at -80°C until required. For microinjection, the crRNA, tracrRNA, Cas9 protein, and a lissamine-labeled control morpholino with no known target gene (Gene Tools, Philomath, OR, USA) were mixed in Yamamoto’s Ringer’s solution (0.75% NaCl, 0.02% KCl, 0.02% CaCl_2_, 0.002% NaHCO_3_) to final concentrations of 100 ng/μl, 100 ng/μl, 400 ng/μl, and 50 nM, respectively. The solution was injected into 1–4-cell-stage zebrafish embryos derived from the Tg (fli1: EGFP) line or albino lines. At 1 dpf, the embryos exhibiting bright lissamine fluorescence were selected and maintained until 6 dpf. At 6 dpf, the selected zebrafish were used for *in vivo* imaging of the ventral artery or were processed for qPCR. After completion of the *in vivo* imaging experiments, genomic DNA was extracted from the zebrafish by incubation in 50 μl of lysis buffer (10 mM Tris-HCl, pH 8.0, 0.1 mM EDTA, 0.2% Triton X-100, 200 μg/ml proteinase K) at 55°C overnight, followed by incubation at 99°C for 10 min. The solution was then placed at 4°C and used as the template for PCR. To detect the crRNA-induced mutations, a short fragment of the ccdc80 gene encompassing the crRNA target sites was amplified from the genomic DNA using ccdc80_gF1 and ccdc80_gR1 primers and QuickTaq (Toyobo, Osaka, Japan). PCR cycling conditions were: 94°C for 2 min followed by 40 cycles of 94°C for 30 s, 60°C for 30 s, and 68°C for 30 s. The PCR products were electrophoresed on 10% poly-acrylamide gel (Wako Chemicals) and visualized by ethidium bromide staining. The crRNA, tracrRNA, and PCR primer sequences are shown in Supplementary Table S3.

### *In Vivo* Imaging of the Ventral Artery of Tg (fli1:EGFP) Zebrafish

Zebrafish at 6 dpf were incubated with or without SNP (1–10 mM) and/or L-NAME (10 mM) in the presence or absence of the protein kinase G (PKG) inhibitor KT5823 (1 μM). Zebrafish were exposed to the chemicals for 1 h for experiments examining the SNP dose-response and the effect of KT5823, and for 2 h for the experiments examining the effect of L-NAME with or without SNP. The zebrafish were then transferred onto glass slides, placed on their backs, and immobilized by covering with a few drops of 3% low-melting point agarose solution. The ventral artery was visualized using an epifluorescence microscope (SMZ25, Nikon, Tokyo, Japan) with GFP-B filters, and images were recorded at 100 frames/s for 5 s. Quantitative assessment of the ventral artery diameter was performed using Volocity (Perkin Elmer, Cambridge, MA, USA). Briefly, the time-lapse images were used to generate M-mode tracings at the level immediately below the first branches of the ventral artery. The ventral artery diameters were then measured manually. The lengths of ventral artery (from the level immediately below the bifurcation to 1st afferent branchial artery to the level immediately above arterial bulb) were also measured manually.

### qPCR Analysis

Total RNA was extracted from control or ccdc80-KO albino zebrafish at 6 dpf using an RNAqueous Micro Kit (Takara, Kyoto, Japan) according to the manufacturer’s protocol. RNA concentrations were determined using a NanoDrop spectrophotometer (Thermo Scientific, Waltham, MA, USA), and aliquots were reverse transcribed using an iScript Select cDNA Synthesis Kit (Bio-Rad, Hercules, CA, USA). qPCR was performed using an ABI Prism 7300 (Life Technologies, Carlsbad, CA, USA) with SYBR Green Realtime PCR Master Mix Plus (Toyobo). The thermal cycling conditions were: 95°C for 1 min followed by 40 cycles of 95°C for 15 s, 60°C for 15 s, and 72°C for 45 s. We measured the expression of collagen type I alpha 1 (*col1a1*), secreted frizzled-related protein 2 (*sfrp2*), endothelin-1 (*edn1*), and glyceraldehyde-3-phosphate dehydrogenase (*gapdh*) mRNA. The data were normalized to *gapdh* mRNA levels to correct for variability in the initial template concentration and the conversion efficiency of the reverse transcription reaction. The primer sequences are shown in Supplementary Table S3.

### Immunohistochemistry

Immunohistochemistry was performed as described previously on lungs excised from control rats or rats with PAH caused by SU5416/hypoxia (Otsuki et al., 2015; Shinohara et al., 2015). Briefly, sections of paraffin-embedded lung tissue were deparaffinized and hydrated. Epitope retrieval was performed by boiling the sections in citrate buffer (0.01 M, pH 6.0), and endogenous peroxidase activity was quenched with 0.3% hydrogen peroxide in methanol. Sections were then blocked in 5% normal goat serum and incubated overnight at 4°C with a rabbit polyclonal antibody to Ccdc80 (ab75881, Abcam, Cambridge, UK) or a mouse monoclonal antibody to α-smooth muscle actin (α-SMA, 1A4; Sigma, St. Louis, MO, USA). Antibody binding was amplified with streptavidin-biotin (LSAB2 kit, Dako, Kyoto, Japan), and sections were incubated with 3,3′-diaminobenzidine substrate and counterstained with hematoxylin. For the negative controls, sections were incubated with rabbit or mouse immunoglobulin instead of the primary antibody (**Figures 5D,H**). The localization and intensity of staining were assessed by two independent examiners (HS and EZ) who were blinded to the rat treatment group.

### Statistical Analysis

Statistical analysis was performed using Prism 6 (GraphPad, La Jolla, CA, USA). Group means were compared by unpaired *t*-test for two groups and by analysis of variance (ANOVA) for four groups. Alpha was set at 0.05, and Dunnett’s multiple comparisons test was used for *post hoc* analysis when significant effects were found by ANOVA. Data are shown as the mean ± standard error (SEM).

## Results

### Identification of DEGs Common to the Five Mammalian PAH Transcriptome Datasets

**FIGURE 1:**
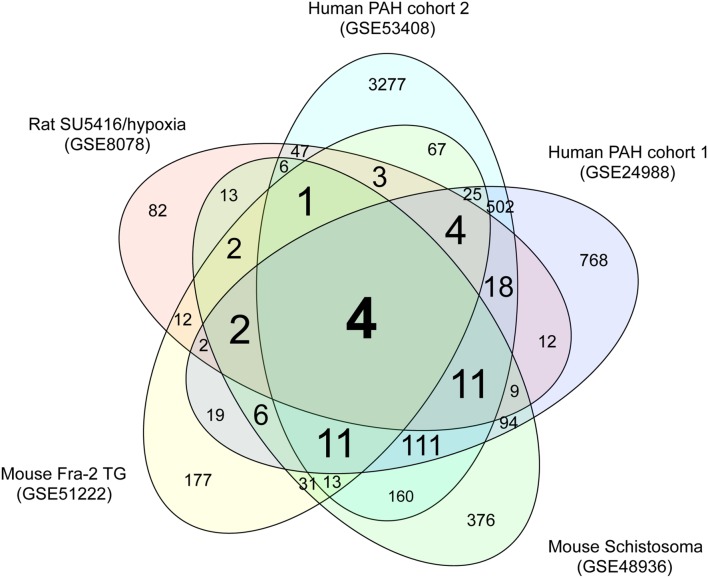
**Venn diagram of DEGs in the five PAH transcriptome datasets.** Transcriptome data from a rat PAH model caused by treatment with the VEGFR inhibitor SU5416 and hypoxia (GSE8078); a mouse PAH model caused by overexpression of Fra-2, a causative gene for systemic sclerosis (GSE51222); a mouse PAH model caused by schistosomiasis (GSE48936); and two cohorts of human PAH patients (GSE24988 and GSE53408) were downloaded from a public database (GEO). Genes that were differentially expressed in the PAH and control groups of each dataset were identified as described in the methods. The numbers of DEGss unique to each transcriptome dataset and shared between datasets are shown.

We performed comparative transcriptome analysis to identify genes dysregulated in the three rodent PAH models and two cohorts of human PAH patients. We identified 228, 379, 850, 1598, and 4260 DEGs in PAH caused by SU5416/hypoxia, Fra-2 TG, schistosomiasis, human PAH cohort 1, and human PAH cohort 2, respectively, compared with the relevant controls (Supplementary Tables S1-1–S1-5). A Venn diagram showing unique and shared DEGs is shown in **Figure 1**. Four DEGs were either upregulated or downregulated in all five datasets (**Table 1**). Expression of coiled-coil domain containing 80 (*CCDC80*) and anterior gradient 2 (*AGR2*) was significantly increased in the five PAH transcriptome datasets, whereas SMAD family member 6 (*SMAD6*) and granzyme A (*GZMA*) were significantly decreased in all datasets. These results suggest that the four DEGs may be novel biomarkers in PAH.

### Identification of Gene Networks Dysregulated in PAH

**FIGURE 2:**
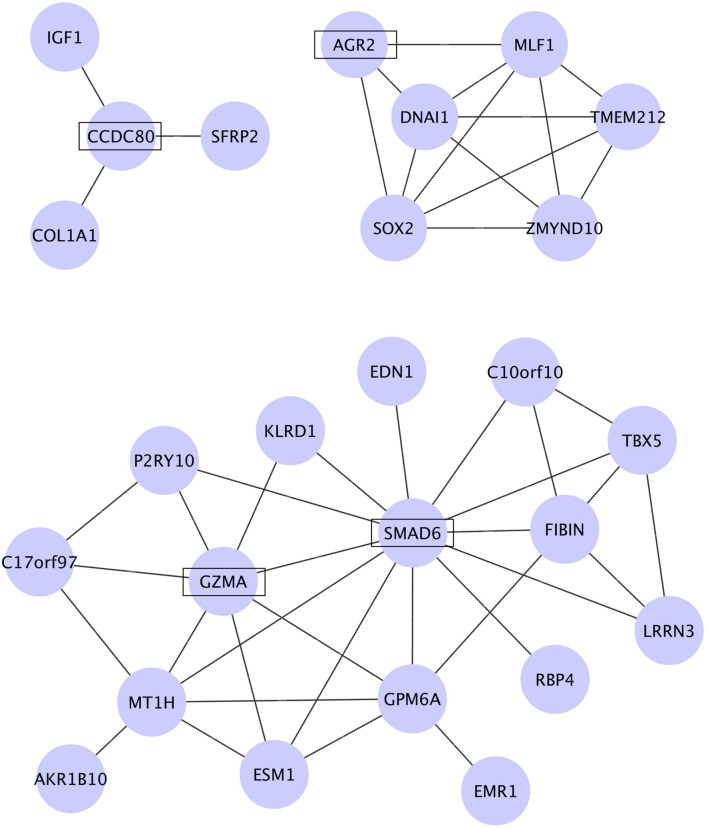
**Networks among the genes dysregulated in PAH identified by WGCNA.** The coefficient of variation (CV) of the normalized probe intensity for each gene in each transcriptome dataset was calculated, and the genes were sorted in descending order by CV. Of the top 3000 genes in each dataset, 40 genes were common to all five datasets and were subjected to WGCNA. The genes were clustered into three modules and the networks identified in these modules are shown. *CCDC80*, *AGR2*, *SMAD6*, and *GZMA*, which were significantly dysregulated in all five PAH transcriptome datasets, are shown in boxes.

To identify molecular networks common to the genes dysregulated in PAH, we used WGCNA. We calculated the coefficient of variation (CV) of the normalized probe intensity for each gene in each transcriptome dataset and then sorted the genes in descending order by CV. The top 3000 genes in each PAH transcriptome dataset were selected (Supplementary Tables S1-1–S1-5). A total of 40 genes were common to the five sets of 3000 genes (Supplementary Tables S2-3–S2-6) and were subjected to WGCNA. **Figure 2** shows the networks assigned to the genes dysregulated in PAH by WGCNA. The four genes identified in **Table 1** (*CCDC80*, *AGR2*, *SMAD6*, and *GZMA*) are included in the three networks identified by WGCNA (**Figure 2**). *CCDC80* is connected to collagen type I alpha 1 (*COL1A1*), secreted frizzled-related protein 2 (*SFRP2*), and insulin-like growth factor 1 (*IGF1*). *SMAD6* is connected to endothelin 1 (*EDN1*) and *GZMA*. *COL1A1*, *IGF1*, and *EDN1* have all been associated with the pathophysiology of PAH (Ooi et al., 2010; Pullamsetti et al., 2014; Madonna et al., 2015; McLaughlin et al., 2015), supporting our hypothesis that *CCDC80*, *SMAD6*, and *GZMA* may be involved in the disease pathophysiology.

### Nitric Oxide Induces Ventral Artery Dilation Through Activation of PKG in Zebrafish

**FIGURE 3:**
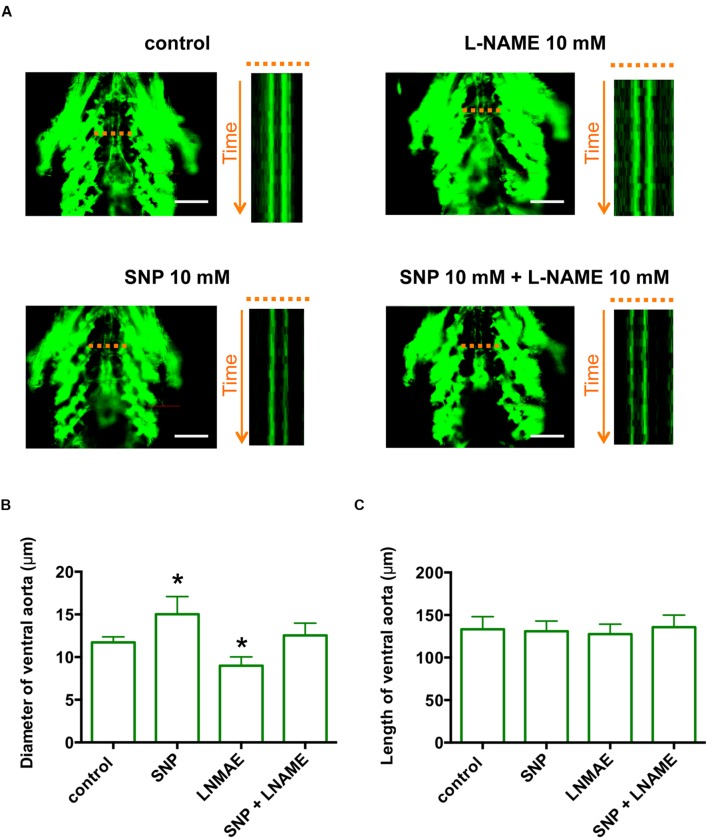
**The zebrafish ventral artery diameter is increased by a NO donor and decreased by a NO synthase inhibitor. (A)** *In vivo* imaging of the ventral artery of Tg (fli1:EGFP) zebrafish at 6 dpf. Zebrafish were incubated with or without 10 mM Sodium nitroprusside (SNP) and/or 10 mM L-NAME for 2 h and then placed on microscope slides on their backs. The ventral artery was imaged under a fluorescent microscope at 100 frames/s for 5 s. The image stack projections and time-lapse imaging traces at the level of the ventral artery (dotted lines) are shown. Scale bars, 100 μm. **(B)** The diameter of the ventral artery was increased and decreased by zebrafish treatment with SNP and L-NAME, respectively, relative to untreated zebrafish. The ventral artery diameter of zebrafish co-treated with SNP and L-NAME was not significantly different from that of control zebrafish. The ventral artery diameters were measured using time-lapse imaging. *N* = 5 for each group. *p < 0.05 vs control. **(C)** The length of ventral artery in zebrafish treated with SNP and/or L-NAME was not significantly different compared to that in untreated zebrafish. *N* = 5 for each group.

Although comparative transcriptome analysis identified *CCDC80* as a novel gene in PAH, the function of *CCDC80* in the vasculature is largely unknown. To analyze the function of *ccdc80 in vivo*, we first established a functionally relevant PAH model using Tg (fli1:EGFP) zebrafish, which express EGFP in vascular endothelial cells (Lawson and Weinstein, 2002). For this, we examined the effect of nitric oxide (NO) on the ventral artery. Treatment of zebrafish with the NO donor SNP dose-dependently dilated the ventral artery (Supplementary Figure S1A), and this was significantly attenuated by co-treatment with KT5823, a specific inhibitor of PKG (Supplementary Figure S1B), indicating that the effects of NO on the ventral artery diameter were regulated though PKG. Inhibition of NO synthase with the specific inhibitor L-NAME significantly decreased the ventral artery diameter when added alone and reversed the artery dilation caused by SNP treatment (**Figure 3**). The length of ventral artery was not significantly different between the groups. These results indicate that, like the pulmonary artery in mammals, the ventral artery in zebrafish is regulated through NO–PKG signaling.

### *ccdc80* Knockout Increases Ventral Artery Diameter and Decreases Expression of *col1a1* and *edn1* in Zebrafish

**FIGURE 4:**
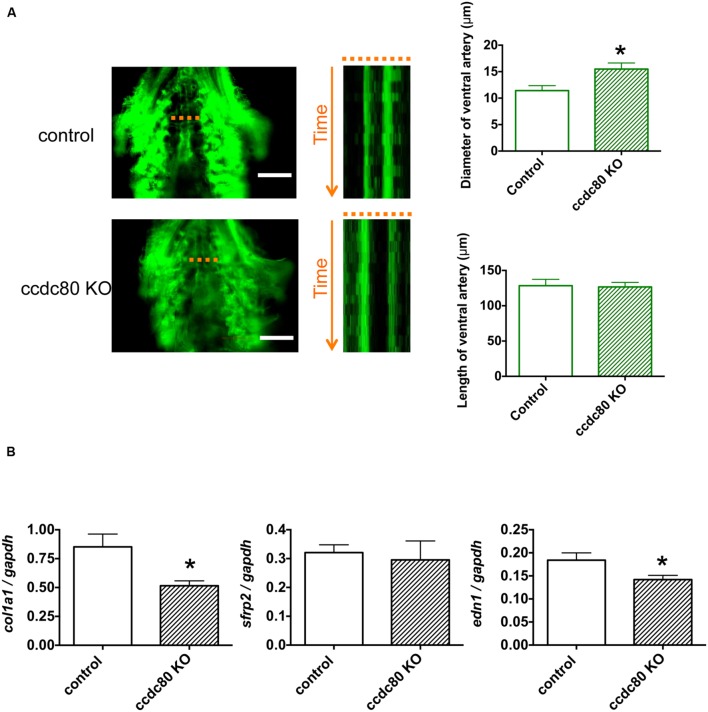
**Knockout of *ccdc80* causes ventral artery dilation and decreases *col1a1* and *edn1* expression in zebrafish. (A)** *In vivo* imaging of the ventral artery of control and ccdc80 knockout Tg (fli1:EGFP) zebrafish at 6 dpf. Zebrafish were placed on slides on their backs, and the ventral artery was imaged under a fluorescence microscope at 100 frames/s for 5 s. Image stack projections and time-lapse imaging traces at the level of the ventral artery (dotted lines) are shown. Bar, 100 μm. The ventral artery diameter of the *ccdc80* knockout zebrafish was significantly larger than that of control, whereas the length of ventral artery was not significantly different between groups. The upper right bar graph shows quantification of ventral artery diameters measured using time-lapse imaging. The lower right bar graph shows quantification of ventral artery lengths. *N* = 6 or 9 for the control and ccdc80 knockout groups, respectively. *p < 0.05 vs. control. **(B)** qPCR analysis of *col1a1*, *sfrp2*, and *edn1* mRNA levels in control and *ccdc80* knockout zebrafish at 6 dpf. Expression of *col1a1*, *sfrp2*, and *edn1* was normalized to that of *gapdh*. *N* = 8 per group. *p < 0.05 vs. control groups.

We next determined whether *ccdc80* plays a role in controlling ventral artery diameter in zebrafish. Previous reports have demonstrated that *ccdc80* is expressed in the cardiovascular system in zebrafish (Brusegan et al., 2012; Della Noce et al., 2015). Using the CRISPR/Cas9 system (Aida et al., 2015; Kotani et al., 2015), we knocked out *ccdc80* in Tg (fli1:EGFP) zebrafish (Supplementary Figure S2). When these animals were examined by fluorescence microscopy, we observed that the ventral artery diameter in *ccdc80*-KO zebrafish was significantly larger than that of control zebrafish (**Figure 4A**). The length of ventral artery was not significantly different between the groups, verifying that the wider diameter of the ventral artery in *ccdc80*-KO zebrafish was not related to the length of the ventral artery in the KO group (**Figure 4A**).

Because WGCNA identified *COL1A1* and *SFRP2* in the *CCDC80* network (**Figure 2**), we compared the whole-body expression of *col1a1* and *sfrp2* mRNA in control and *ccdc80*-KO zebrafish. As shown in **Figure 4B**, the expression of *col1a1* mRNA was significantly lower in ccdc80-KO animals compared with the controls, whereas *srfp2* mRNA levels were unchanged. *COL1A1* expression is increased by overexpression of *EDN1* (Hirt et al., 2012) and decreased by inhibition of *EDN1* synthesis (Cowling, 2015); therefore, we also measured the effect of *ccdc80* deletion on *edn1* expression. We found that *edn1* mRNA levels were significantly lower in *ccdc80*-KO zebrafish compared with the control animals (**Figure 4B**). These results suggest that CCDC80 may regulate the pulmonary artery tone by modulating endothelin-1-induced collagen expression.

### Ccdc80 Expression Is Increased in the Pulmonary Vascular Lesion in a Rat PAH Model

**FIGURE 5:**
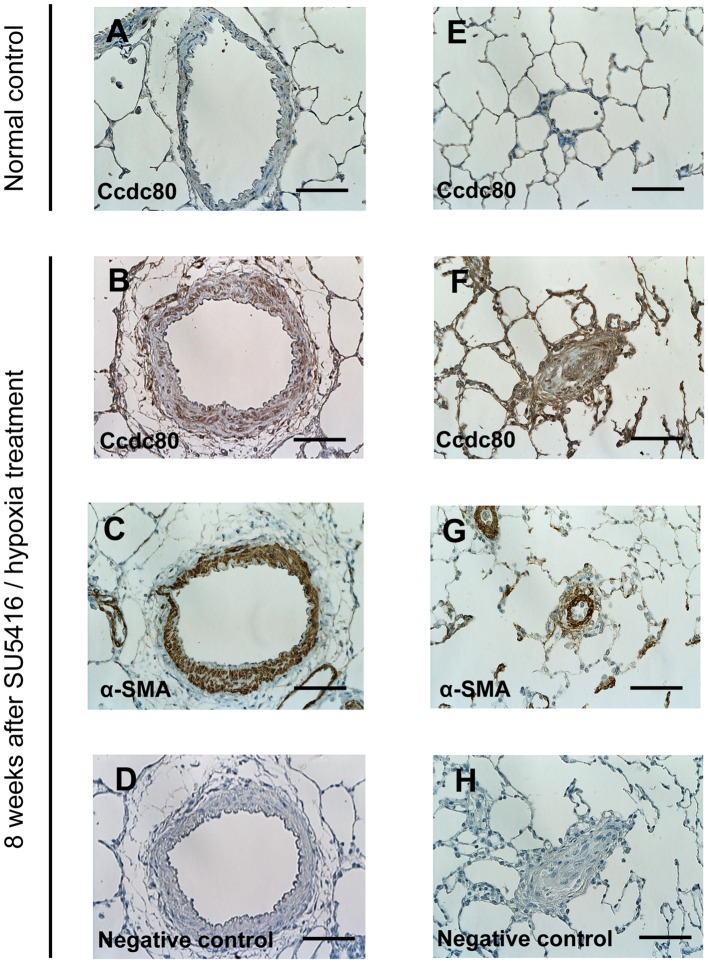
**Expression of Ccdc80 is increased in the lungs of rats with PAH. (A–H)** Representative photomicrographs of pulmonary arteries (PAs) from control rats **(A** and **E)** or rats with PAH induced by SU5416 /hypoxia **(B–D** and **F–H)**. Serial cross-sectional views of pre-acinar PA **(A–D)** and intra-acinar PA **(E–H)** immunostained for Ccdc80 and α-smooth muscle actin (α-SMA). Scale bars, 50 μm. Original magnification (×400).

To establish the significance of our findings for PAH pathology, we performed immunohistochemical staining of Ccdc80 protein in the lung tissues of control rats and rats with PAH caused by SU5416 and hypoxia. Ccdc80 was not readily detected in the PAs of control rats (**Figures 5A,E**), but there was intense immunoreactivity in the hypertrophied media and adventitia of the pre-acinar PAs (**Figure 5B**) and in the thickened intima, media, and adventitia of the obstructed intra-acinar PAs (**Figure 5F**) in the PAH rat. The Ccdc80 immunoreactivity was localized in the α-SMA–positive cells in the PA media but not in the intima or adventitia (**Figures 5C,G**).

## Discussion

### Involvement of *CCDC80*, *SMAD6*, *AGR2*, and *GZMA* in PAH

We demonstrated that expression of *CCDC80*, *SMAD6*, *AGR2*, and *GZMA* was significantly dysregulated in two cohorts of human PAH patients (Mura et al., 2012; Zhao Y. et al., 2014; Zhao Y.D. et al., 2014) and in three rodent PAH models caused by: (i) treatment with a VEGF receptor inhibitor under conditions of hypoxia (Moreno-Vinasco et al., 2008); (ii) overexpression of Fra-2, a causative gene for systemic sclerosis (Biasin et al., 2014); and (iii) schistosomiasis (Graham et al., 2013).

CCDC80, which was significantly upregulated in the five PAH transcriptome datasets, is a 950-amino acid secreted protein that binds to ECM proteins, including glycosaminoglycans, and promotes cell adhesion (Manabe et al., 2008). Interestingly, human steroid-sensitive gene 1 (SSG1), which is identical to the 530-amino acid amino-terminal sequence of human CCDC80 protein, is highly expressed in cardiovascular systems and is phosphorylated by PKG (Wang et al., 2013). These studies suggest that *CCDC80* may regulate vascular function via the ECM and PKG signaling. Consistent with this, we demonstrated that knockout of *ccdc80* in zebrafish caused dilation of the ventral artery and decreased the expression of *col1a1*. We also found that pharmacological inhibition of PKG significantly attenuated the effect of *ccdc80* knockout on the zebrafish ventral artery (data not shown). Collectively, these findings point to a possible role for *CCDC80* in the pathogenesis of PAH (discussed later).

*SMAD6*, a member of the SMAD family of TGFβ signaling regulators (Li, 2015), was also downregulated in all five PAH transcriptome datasets. SMAD6 has been shown to negatively regulate TGFβ signaling through the TGFβ-activated kinase (TAK1)–mitogen-activated protein kinase (MAPK) pathway (Jung et al., 2013). Heterozygous mutations in the type II receptor for bone morphogenetic protein (*BMPR2*), which underlie the majority of the inherited and familial forms of PAH (Guignabert et al., 2015), stimulate the TGFβ–TAK1–MAPK pathway (Nasim et al., 2012). These studies suggest that reduced expression of SMAD6 may increase TGFβ–TAK1–MAPK pathway activity, similar to the effects of BMPR2 mutation. Because inhibition of the TGFβ–TAK1–MAPK pathway rescues abnormal proliferation and apoptosis of pulmonary artery smooth muscle cells isolated from *BMPR2* mutant mice (Nasim et al., 2012), normalizing the decrease in *SMAD6* expression may be therapeutic in PAH.

The expression of *AGR2* was significantly increased in the five PAH transcriptome datasets. AGR2 has been proposed to be involved in development, tissue regeneration, and cancer metastasis (Brychtova et al., 2011). AGR2 can bind to α-dystroglycan, which regulates the ECM and interaction with integrins (Edeleva and Shcherbata, 2013). Thus, the increase in *AGR2* expression may be related to the dysregulated ECM observed in PAH.

*GZMA* was significantly downregulated in all five PAH transcriptome datasets. GZMA is a member of the granzyme family of proteins, which are important mediators of cell death induced by immune cells (Hendel et al., 2010). Previous work has shown that TGFβ decreases *GZMA* expression and inhibits its function in cytotoxic T cells (Thomas and Massague, 2005). Dysregulation of cytotoxic T cells has been reported in PAH (Ulrich et al., 2008; Savai et al., 2012), raising the possibility that the decrease in *GZMA* expression in PAH may be related to the dysregulation of cytotoxic T cells.

In addition to these observations, the expression levels and/or activities of CCDC80, AGR2, and GZMA in human plasma have been related to various pathological states (Accardo-Palumbo et al., 2010; Kani et al., 2013; Menni et al., 2015). It may therefore be worthwhile to perform similar measurements of these proteins in the plasma of human PAH patients and determine whether their levels and/or activities correlate with the clinical stages of PAH.

### CCDC80 May Regulate Vascular Tone by Modulating Endothelin-1–induced Collagen Expression

In this study, we demonstrated that knockout of *ccdc80* in zebrafish caused dilation of the ventral artery and decreased the expression of both *edn1* and *col1a1*. We also showed that Ccdc80 immunoreactivity was increased in the hypertrophied media and adventitia of the pre-acinar PAs and in the thickened intima, media, and adventitia of the obstructed intra-acinar PAs in a rat PAH model.

The expression of *CCDC80* was correlated with fibrillin-1 whose expression is induced by TGFβ (Summers et al., 2010), suggesting that it may be induced by TGFβ signaling. TGFβ signaling is activated in familial PAH caused by mutation of *BMPR2* and in syndromic PAH caused by systemic sclerosis or schistosomiasis (Varga and Pasche, 2009; Aschner and Downey, 2016). In various models, PAH is ameliorated by blockade of TGFβ signaling through diverse mechanisms, including administration of neutralizing antibodies, antisense nucleotides, or TGFβ receptor kinase inhibitors, and by gene transfer of inhibitory SMAD (Varga and Pasche, 2009; Aschner and Downey, 2016). TGFβ signaling regulates vascular tone, including that of PAs, by regulating the expression of vasodilators such as NO and vasoconstrictors such as endothelin-1 (Pardali and ten Dijke, 2012). These studies suggest that expression of *CCDC80* may positively correlate with TGFβ signaling, leading to decreased NO and increased endothelin-1 levels. Further studies using both *in vitro* and *in vivo* approaches are required to fully elucidate the functions of CCDC80 in the pathophysiology of PAH and to examine its potential as a marker and/or a therapeutic target in PAH.

## Author Contributions

YN conceived the study, performed the bioinformatics analyses, and wrote the manuscript. SS performed the analysis of zebrafish. HS and EZ developed the rat PAH model, performed the immunohistochemical analysis of Ccdc80 expression, and wrote the manuscript. SO, SM, YA, MY, KK, and RK performed the experiments. YM and KM developed the rat PAH model and wrote the manuscript. TT conceived the study and wrote the manuscript.

## Conflict of Interest Statement

The authors declare that the research was conducted in the absence of any commercial or financial relationships that could be construed as a potential conflict of interest.

## Abbreviations

DEGs: differentially expressed genes
dpf: days post-fertilization
ECM: extracellular matrix.
L-NAME: *N*-omega-nitro-L-arginine methyl ester
PAH: pulmonary artery hypertension
PKG: cGMP-dependent protein kinase
SNP: sodium nitroprusside.
