# Supplementary figures and images for: Comparative Transcriptome Analysis Identifies CCDC80 as a Novel Gene Associated with Pulmonary Arterial Hypertension
Source: Front Pharmacol. 2016 Jun 7;7:142. doi: 10.3389/fphar.2016.00142 (PMC4894905; doi:10.3389/fphar.2016.00142)

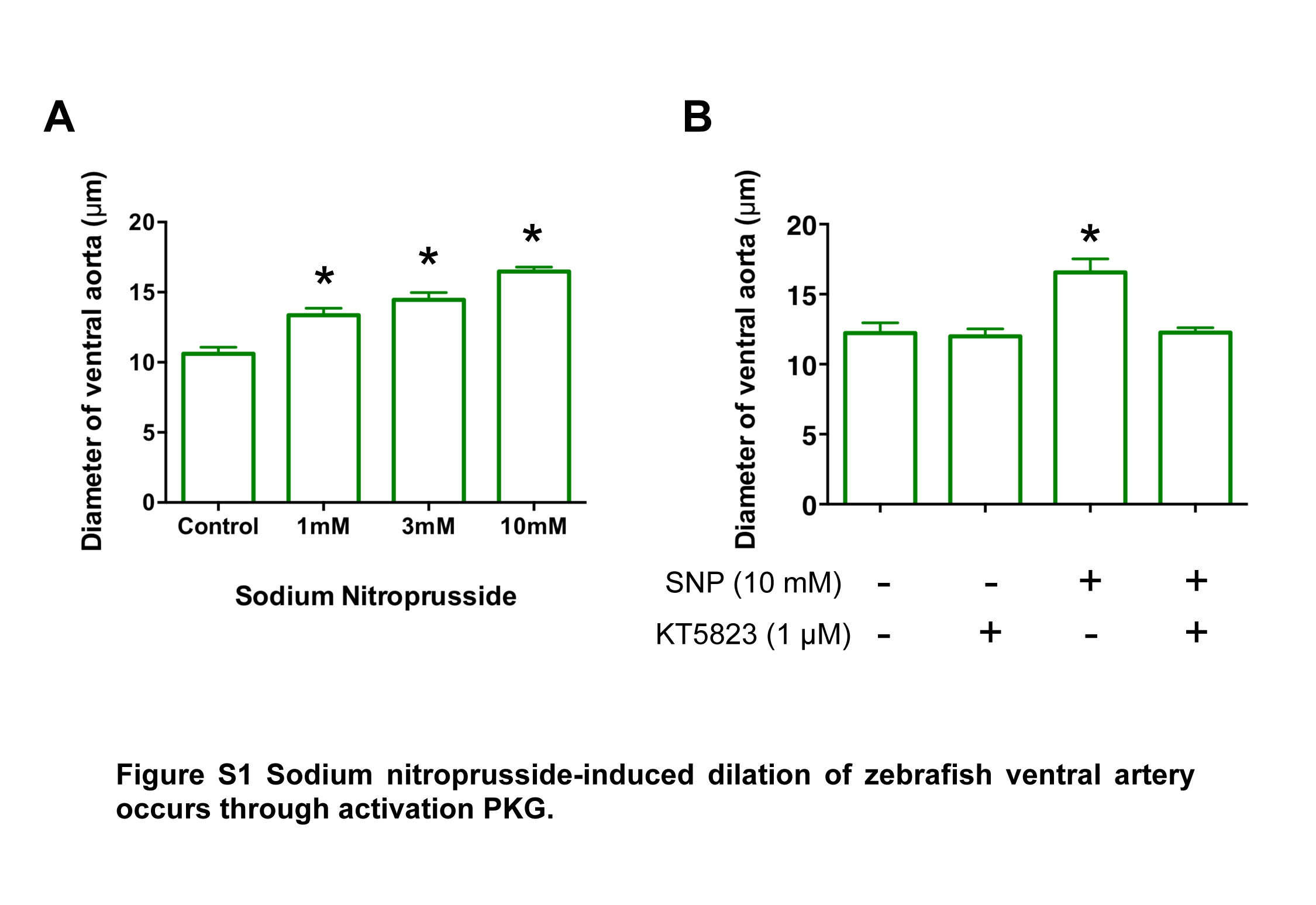

Supplement: Supplementary file 1 [file Image_1.JPG]

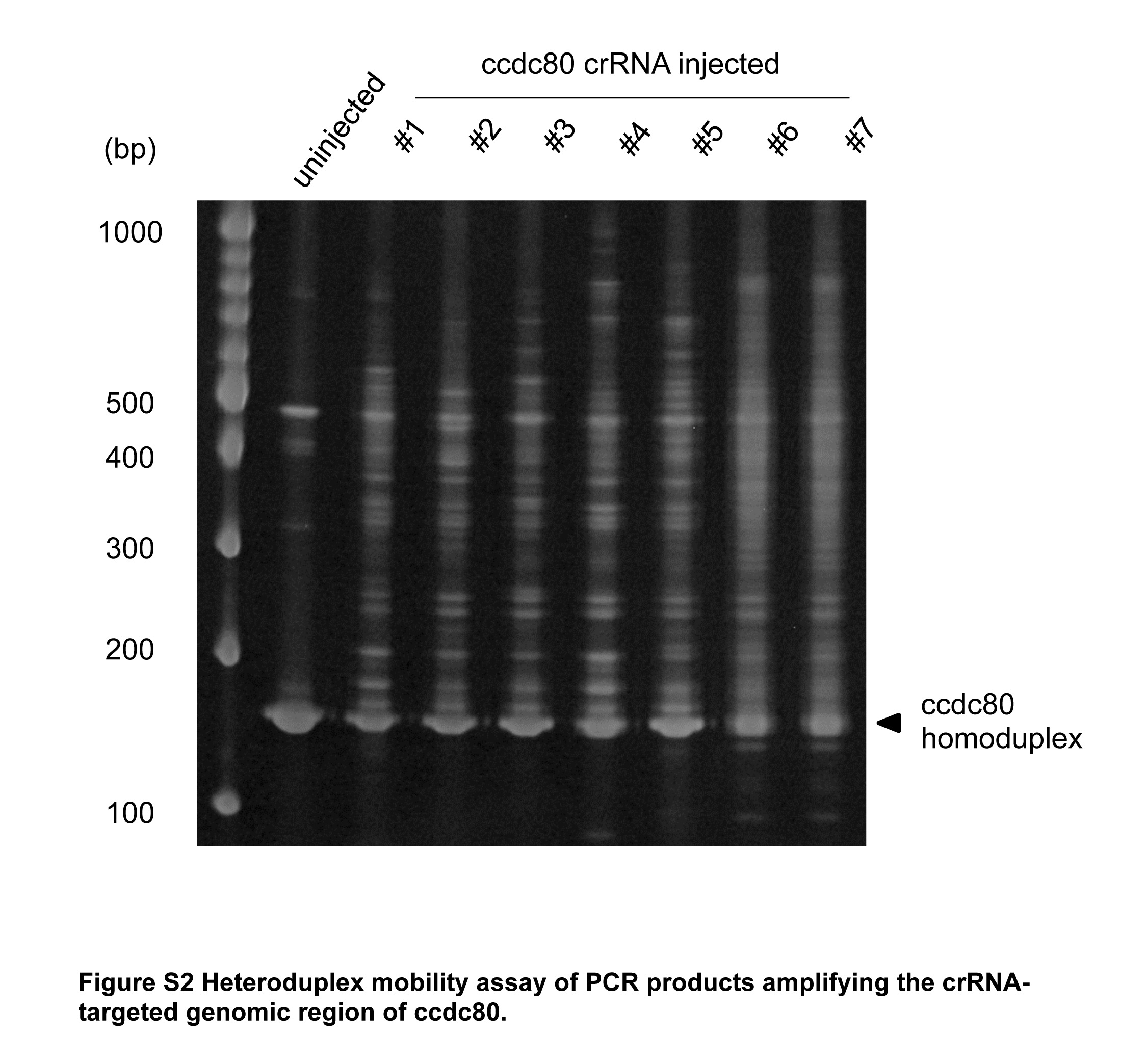

Supplement: Supplementary file 2 [file Image_2.JPG]
